# Supplementary figures and images for: Wastewater surveillance overcomes socio-economic limitations of laboratory-based surveillance when monitoring disease transmission: The South African experience during the COVID-19 pandemic
Source: PLoS One. 2025 Feb 25;20(2):e0311332. doi: 10.1371/journal.pone.0311332 (PMC11856519; doi:10.1371/journal.pone.0311332)

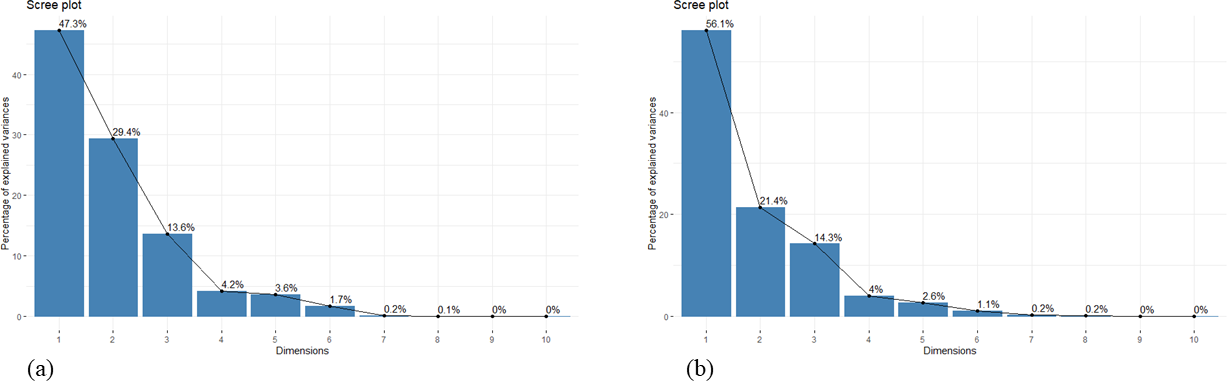

Supplement: S1 Fig — Dimension 1 and 2 account for 76.7% in sewershed D (a) and 77.5% sewershed O (b). (TIF) [file pone.0311332.s001.tif]

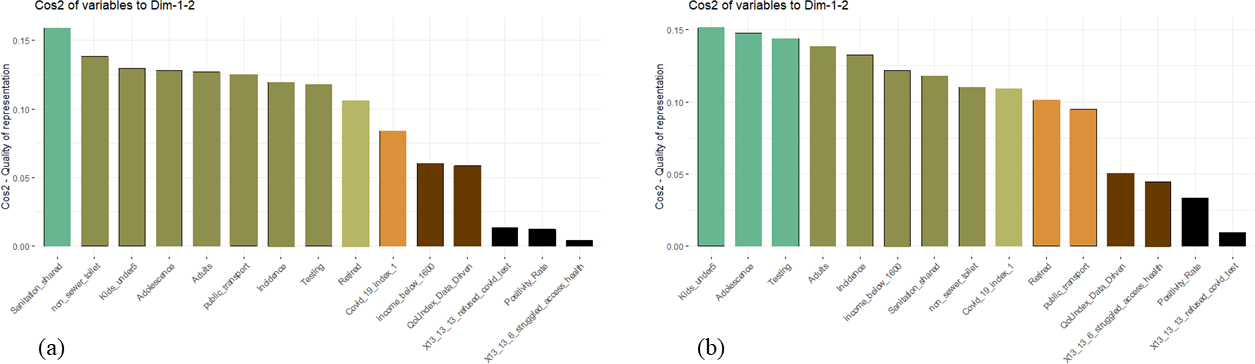

Supplement: S2 Fig — Note: Colour ramp matches the PCA plots and the darker the shade the lower the loading value. (TIF) [file pone.0311332.s002.tif]
